# Differential Activity of Type I Interferon Subtypes for Dendritic Cell Differentiation

**DOI:** 10.1371/journal.pone.0058465

**Published:** 2013-03-05

**Authors:** Geneviève Garcin, Yann Bordat, Paul Chuchana, Danièle Monneron, Helen K. W. Law, Jacob Piehler, Gilles Uzé

**Affiliations:** 1 Unité Mixte de Recherche 5235, Centre National de la Recherche Scientifique, University of Montpellier II, Montpellier, France; 2 Institut des Neurosciences de Montpellier, Institut National de la Santé Et de la Recherche Médicale 844, Montpellier, France; 3 Centre for Human Immunology, Institut Pasteur, Paris, France; 4 Division of Biophysics, University of Osnabrück, Osnabrück, Germany; Oklahoma Medical Research Foundation, United States of America

## Abstract

The type I interferon (IFN) family comprises 15 cytokines (in human 13α, 1β, 1ω), which exert several cellular functions through binding to a common receptor. Despite initial activation of the same Jak/Stat signalling pathway, the cellular response may differ depending on type I IFN subtype. We investigated the activity of six type I IFN subtypes - IFNα1, α2, α8, α21, ω and β- to promote the differentiation of dendritic cells (DC). Transcriptome analyses identified two distinct groups, the IFNα/ω-DC and the IFNβ-DC. In addition, the expression level of seven chemokines and several cell surface markers characteristic of DC distinguished IFNα-DC and IFNβ-DC. These differences are unlikely to impact the efficacy of T cell functional response since IFNα2-DC and IFNβ-DC were equipotent in inducing the proliferation and the polarization of allogenic naïve CD4 T cells into Th1 cells and in stimulating autologous antigen specific CD4 or CD8 T cells. Of the functional parameters analysed, the only one that showed a modest differential was the phagocytic uptake of dead cells which was higher for IFNα2-DC.

## Introduction

The type I interferon (IFN) family is composed of several cytokines, resulting in a high level of complexity in mammals. In humans, for example, there are 13α, 1β, 1ω, and two other poorly characterized subtypes, which all act through a common receptor and primarily activate the same Jak/Stat signalling pathway [1]. The α/β/ω subtypes are produced by many cell types in response to pathogen infection and, in addition to their antiviral and antiproliferative activities, play key roles in the onset of innate and adaptive immune responses by regulating cell differentiation, death and survival. In the absence of any infection low amounts of type I IFN are expressed in many tissues to maintain homeostasis in the immune cell network [2]. In particular, the action of endogenous type I IFN on dendritic cells (DC) is required for tumor immunosurveillance [3], [4].

For a given biological activity, the potency of individual α/β/ω subtypes can vary considerably, and several studies have reported differential activities of type I IFNs. For example, the α2 and β subtypes exhibit the same specific antiviral activity, show discrete differences in their potencies to activate the Jak/Stat signalling pathway and to induce the transcription of early ISGs, while IFNβ is much more potent at inhibiting cell growth or osteoclastogenesis [5], [6]. Even if the molecular basis of differential activities among type I IFN subtypes is becoming more clear [6], [7], [8], [9], [10], [11], the physiological relevance of this phenomenon remains elusive. The fact that, in human populations, some subtypes appear to be under purifying selection, whereas others are likely undergoing pseudogenization suggests that they are not equivalent in terms of function [12]. In clinical practice, it is interesting to note that the antitumor efficacy of administered IFNα2 is associated with the development of autoimmune manifestations [13], whereas IFNβ is routinely used for treatment of multiple sclerosis, which is considered to be an inflammatory autoimmune disease [1].

The action of type I IFN on DC is important for both the natural process of tumor immunosurveillance and the antitumor action of therapeutically administered IFN [14]. Thus, the aim of this study was to establish whether human type I IFN subtypes exert differential activities on the functions and differentiation of DC. Type I IFN is a strong inducer of monocyte differentiation into highly activated and partially mature DC that can internalize antigen and efficiently prime T cell responses [15], [16], [17]. This IFN activity could reflect in part the mechanism by which type I IFN exerts an essential role in controlling tumor immune response and acts as an antitumor agent when therapeutically administered to patients [3], [4], [14], [18], [19].

In this paper, we have compared the activities of four IFNα subtypes, IFNβ and IFNω to induce the differentiation of human monocytes into IFN-DC. Whereas the IFN subtypes studied result in DC equally effective at driving Th1 cell development, IFNβ-differentiated DC differ by their unique gene expression profile, chemokine synthesis and by reduced ability to phagocytose apoptotic and necrotic dead cells. This study emphasizes the particularity of IFNβ among the other type I IFN subtypes.

## Results

### IFNα/ω-DC and IFNβ-DC show differential gene expression profile

Monocytes were differentiated into DC using GM-CSF and different type I IFN subtypes. In order to investigate effects not caused by different specific activities in inducing early transcriptional response, IFN concentrations were adjusted as described in the materials and methods section.

**Figure 1:**
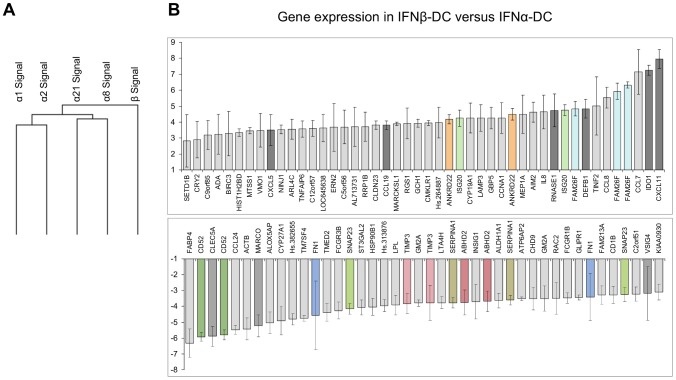
IFNα and IFNβ are not equivalent in inducing the differentiation of human blood monocytes into dendritic cells (IFN-DC). Gene expression profiles of IFNα1, α2, α8, α21 and IFNβ-DC were analyzed on HG-U133 Plus 2.0 microarrays. A. Hierarchical clustering of IFN-DC. B. Genes differentially expressed between IFNα-DC and IFNβ-DC. Results are expressed as median ± SD. In color, genes identified as differentially expressed by 2 or more probe sets. In dark grey genes subsequently analyzed by RT-qPCR in DC differentiated from monocytes isolated from several blood donors.

First, the transcriptomes of IFNα1, α2, α8, α21 and β-derived DCs differentiated from monocytes isolated from the same individual were investigated by using HG-U133 Plus 2.0 Affymetrix microarrays. In order to identify correlated gene expression patterns, unsupervised hierarchical clustering was applied to microarray data meeting the following criteria: the gene must be present in a least 80% of the samples and the deviation standard of the signal must be superior to 10%. Hierarchical clustering was applied to one axe (arrays) and performed with the Pearson uncentered correlation as similarity metric and average linkage as clustering method. As reflected by the dendrogram, the samples displayed heterogeneous expression profiles and were sorted into two major groups showing differential expression profiling, the IFNα-DC group on one side and the IFNβ-DC on the other side (Fig. 1A). Other parameters for the similarity metric (Pearson centered correlation and Euclidian distance) were applied to the data set for the generation of the dendrogram, but whatever the parameters used, the tree of classification obtained was the same, indicating a robustness of the result.

Based on the expression similarity presented by the dendrogram, data were analyzed according to the EV method [20]. 54,642 probsets common to the four IFNα-DC were first sorted and their expression levels were compared with expression in the IFNβ-DC sample. This allows the identification of 78 genes differentially expressed between IFNα-DC and IFNβ-DC, 42 being more expressed in IFNβ-DC and 36 more expressed in IFNα-DC group (Fig. 1B). Interestingly, many of the differentially expressed genes are important players in DC biology, including several chemokines, such as *CXCL11*, some receptors involved in the process of phagocytosis (*FCGR*, *MARCO*, *CLEC5A*), a defensin (*DEFB1*) and *IDO1* described to be implicated in induction of tolerance [21].

**Figure 2:**
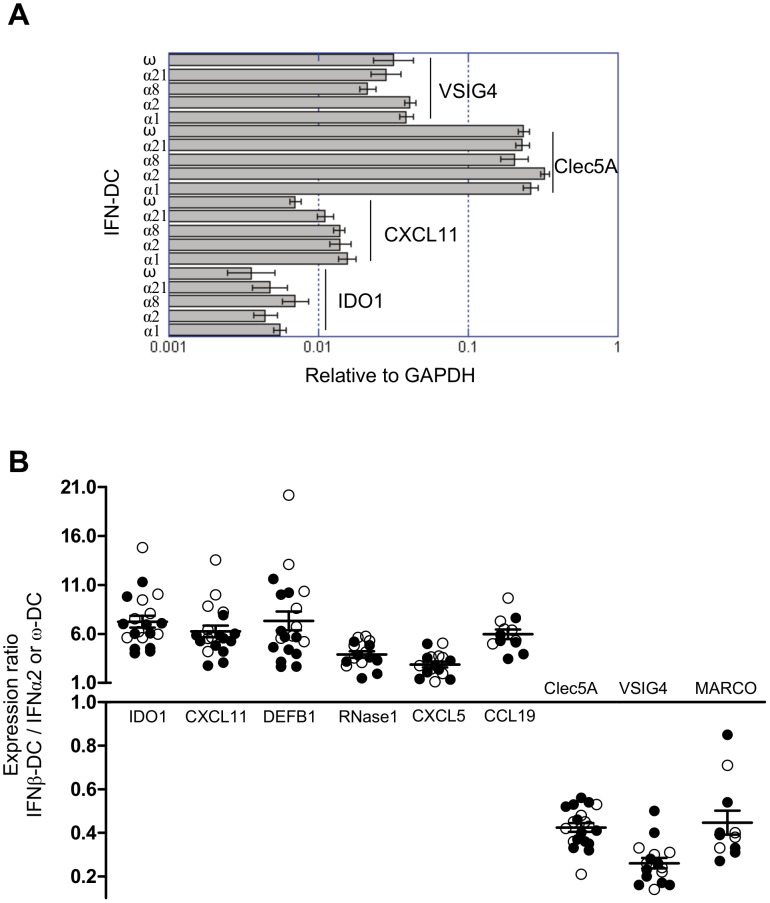
Analysis of gene expression by RT-qPCR. **A**. IFNα1, α2, α8, α21 and ω-DC were generated from monocytes from three independent donors and gene expression level of CXCL11, VSIG4, Clec5A and IDO1 analyzed by RT-qPCR. Expression level is relative to GAPDH. One experiment representative of the three is shown. **B**. IFNα2, ω and β-DC were generated from monocytes from 5 to 11 independent donors and 9 genes were analyzed by RT-qPCR for their expression. Results are expressed as a ratio: (gene expression in IFNβ-DC/GAPDH)/(gene expression in IFNα-DC or IFNω-DC/GAPDH). Closed symbols are for IFNα2-DC and open symbols for IFNω-DC.

In a second step, in order to test the reproducibility of the data described in Fig. 1B across DC differentiated from several individuals, a set of genes was chosen for their relevance to DC biology, and levels of expression were analyzed by RT-qPCR. Monocytes from three independent donors were differentiated into DC with IFNα1, α2, α8, α21 or ω, and expression of *VSIG4*, *CXCL11*, *CLEC5A* and *IDO1* was measured. The results obtained confirmed that all IFNα subtypes are equipotent in regulating the expression of these genes and showed that the IFNω-DC is closely related to IFNα-DC group for this gene set (Fig. 2A). This result was reinforced by qPCR analyses performed on IFNα2, ω and β-DC from 5 to 11 independent blood donors and for 9 genes (Fig. 2B). Indeed, 6 genes (*IDO1*, *CXCL11*, *DEFB1*, *RNAse1*, *CXCL5* and *CCL19*) were found higher expressed in IFNβ-DC by a factor of 6.24±0.63, 5.62±0.58, 6.79±1.15, 3.75±1.27, 2.81±0.42 and 5.30±0.85, respectively and 3 genes (*CLEC5A*, *VSIG4* and *MARCO*) higher expressed in IFNα2/ω-DC by a factor of 2.42±0.15, 4.29±0.42 and 2.36±0.35 respectively. Overall, there was a good concordance between results obtained from microarray and those obtained by RT-qPCR.

### IFNβ-DC express higher levels of membrane markers associated with IFN-DC differentiation status and produce greater amounts of chemokines

Since Affymetrix and qPCR data strongly supported that IFNω belongs to the IFNα group, subsequent work focused on the two clinically used type I IFN: IFNα2 and IFNβ-DC.

IFN-DC were analysed for expression of cell surface markers commonly used to assess the differentiation of DC. As shown in Table 1 and previously described [22], a 3-day exposure of monocytes to type I IFN results in up-regulation of the co-stimulatory molecules CD80, CD86, CD40 and HLA-DR and down-regulation of CD14 in both types of IFN-DC. The comparison between IFNα2-DC and IFNβ-DC showed that CD14 was downregulated to a greater degree in IFNβ-DC than in IFNα2-DC. For all others markers, except percentage of HLA-ABC and HLA-DR positive cells, a differential was observed between IFNα2-DC and IFNβ-DC (Table 1). These statistically significant differences are minor, however, and their impact on the biological function of DC are not established.

**Table 1:** Expression of cell surface markers characteristic of DC differentiation state.

	Fluorescence median**				% positive cells**			
Cell surface markers	Monocytes*	IFNα2-DC	IFNβ-DC	Differential α2, β : p value***	Monocytes*	IFNα2-DC	IFNβ-DC	Differential α2, β : p value***
**CD14**	1785.0±184.4	602.0±54.6	334.3±27.3	<0.0001	78.6±1,6	61.8±2,6	46,9±2,7	<0.0001
**CD83**	37.5±3.4	51.2±4,9	70.2±7,3	<0.0001	9.2±1.1	10.0±1.0	14.4±1.5	<0.0001
**CD80**	124.8±20.1	689.7±52.7	944.7±109.8	<0.0001	20.7±2.9	72.6±1.9	76.0±1.8	<0.0001
**CD86**	832.0±107.8	2425.0±281.2	3292.0±386.6	<0.0001	67.2±3.3	87.1±1,5	88.5±1.5	0,0013
**CD40**	949.0±130.0	2114.0±274.5	2703.0±359.2	<0.0001	72.4±2.8	86.5±1.5	85.4±1.5	0,0123
**HLA-ABC**	824.7±59.0	2182.0±142.2	2341.0±134.2	0,0026	78.1±1.8	94.7±0.5	94.1±0.7	0,0649
**HLA-DR**	4513.0±280.8	5360.0±317.1	6650.0±386.3	<0.0001	85.9±1.6	90.4±1.1	89.6±1.2	0,0836

* Monocytes cultured during 3 days with GMCSF and autologous plasma but without IFN.

** n = 53 on 26 independent blood donors.

*** Wilcoxon matched pairs test.

**Figure 3:**
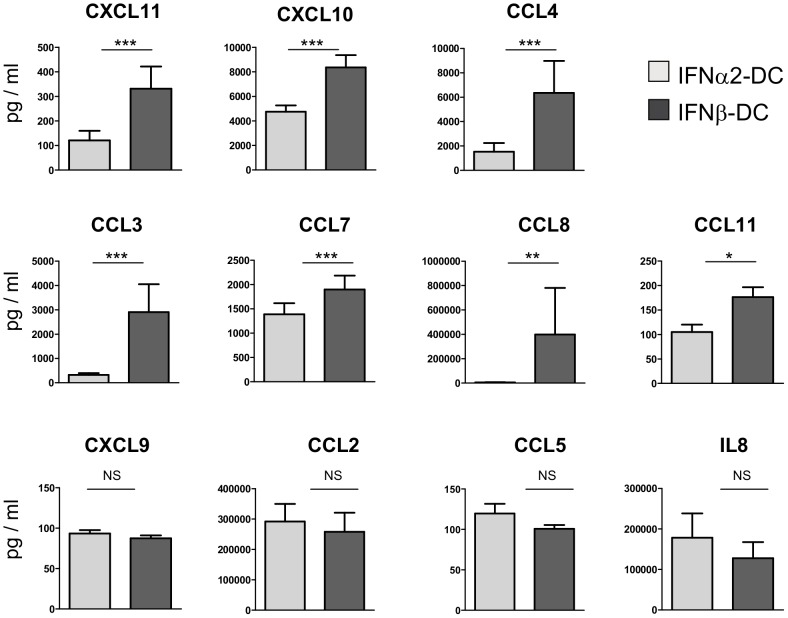
IFNβ-DC produce higher amounts of chemokines compared to IFNα2-DC. After 3 days of differentiation, IFN-DC supernatants were collected and amount of chemokines produced quantified by luminex. Error bars represent the SEM. P value was determined using a Wilcoxon matched pairs test. Experiments were done on cells from nine independent blood donors for CXCL11, CXCL10, CCL4, CCL3, CCL7, CXCL9, CCL2, CCL5 and IL8, eight independent blood donors for CCL8 and six independent blood donors for CCL11. *** indicates P<0.005, ** P<0.01 and * P<0.05. NS: not significant.

The quantification of chemokine production by IFN-DC also highlights the difference between the two types of DC. Among all chemokines analyzed, CXCL11, CXCL10, CCL4, CCL3, CCL7, CCL8 and CCL11 were found to be more produced by IFNβ-DC than by IFNα2-DC, none were more produced by IFNα2-DC and CXCL9, CCL2, CCL5 and IL8 were produced in equal amounts (Fig. 3).

### Functional comparison of IFNα2-DC and IFNβ-DC for T cell stimulation, migration and phagocytosis of dead cells

**Figure 4:**
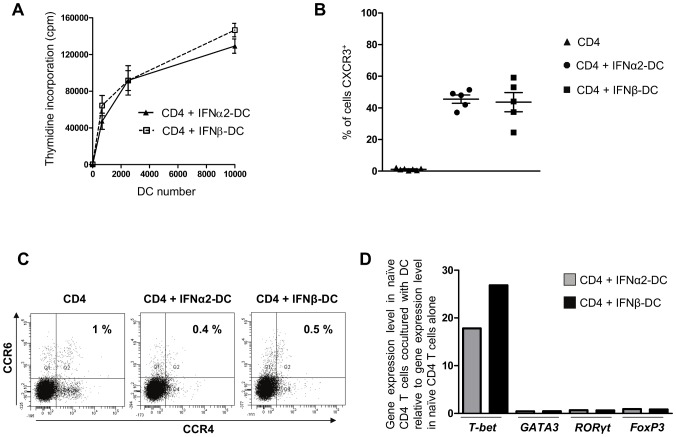
IFNα2-DC and IFNβ-DC are equally effective in inducing proliferation and differentiation of CD4^+^ T cells. **A**. Mixed lymphocyte reaction assay in which IFN-DC were used as stimulators. Proliferation of allogenic CD4^+^ T cells was determined after 6 days of co-culture by measuring thymidine incorporation. Data are from three independent experiments performed on cells from three independent blood donors and error bars indicate SEM. **B–C**. Naïve allogenic CD4^+^ T cells were cocultured with IFNα2-DC or IFNβ-DC for 6 days and differentiated T cells were analyzed by FACS for the expression of CXCR3 (**B**), CCR4 and CCR6 (**C**). In B, experiments were performed on cells from five independent blood donors. Error bars indicate SEM. In C, one experiment carried out on cells from a single blood donor and representative of five experiments done on five independent donors is shown. **D**. RT-qPCR analysis of *T-bet, GATA3, RORγt* and *FoxP3* mRNA levels in differentiated T cells after coculture of naïve CD4^+^ T cells with IFN-DC for 6 days. Results are expressed as a ratio: gene expression level in naïve CD4^+^ T cells cultured with DC relative to gene expression level in naïve CD4^+^ T cells alone. Data are from samples from one of the five independent experiments reported in panel B.

The functionality of the *ex-vivo* generated IFN-DC was tested using an allogenic mixed lymphocyte assay (MLR). As shown in Fig. 4A, IFNα2-DC and IFNβ-DC were found to be equally effective in inducing the proliferation of CD4^+^ T cells.

Similarly, IFNα2-DC and IFNβ-DC were able to polarize cord blood-derived naïve CD4^+^ T cells in the same way. Indeed, 45.48%±2.62 and 43.58%±6.07 of naïve CD4^+^ T cells became positive for CXCR3, indicating a Th1 phenotype, when co-cultured with IFNα2-DC and IFNβ-DC, respectively (Fig. 4B). In addition, both IFN-DC failed to polarize naïve T cells into Th17 as was assessed, by measuring the co-expression of surface CCR4 and CCR6, in Fig. 4C. Accordingly, measurement, by RT-qPCR, of *T-bet, GATA3, RORγt* and *FoxP3* mRNA expression levels in T cells sorted from the MLR indicate that both IFNα2-DC and IFNβ-DC are able to polarize naïve CD4^+^ T cells only towards the Th1 phenotype (Fig. 4D).

**Figure 5:**
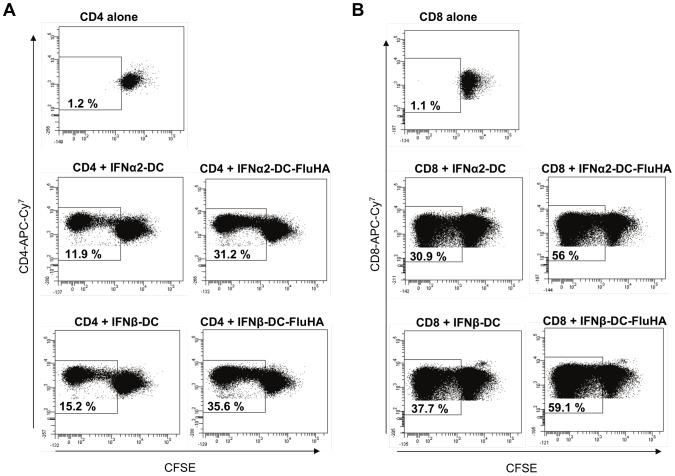
IFNα2 and IFNβ-DC loaded with Flu-HA protein induce the proliferation of autologous T cells with the same potency. IFN-DC were pulsed or not (control) with Flu-HA for 24 h and were then cocultured with autologous CFSE-labelled CD4^+^ (**A**) or CD8^+^ (**B**) T cells at a ratio of 1∶5 in AIM-V medium. T cells were analysed by FACS 5 days later for CFSE dilution after gating on viable (7-AAD^−^) CD4^+^ or CD8^+^ T cells. One experiment representative of six, carried out on five independent donor samples and of four experiments carried out on four independent donors are shown for CD4 and CD8 T cells, respectively.

In order to investigate the capacity of IFN-DC to re-activate autologous antigen specific T cells, IFN-DC were pulsed with influenza protein HA prior to co-culture with either autologous CFSE-labeled CD4^+^ T or CD8^+^ T cells. Results indicate that IFNα2-DC and IFNβ-DC induced the proliferation of CD4^+^ (19.3 and 20.4% respectively; Fig. 5A) and CD8^+^ T cells (25.1 and 21.4% respectively; Fig. 5B) with the same efficiency.

**Figure 6:**
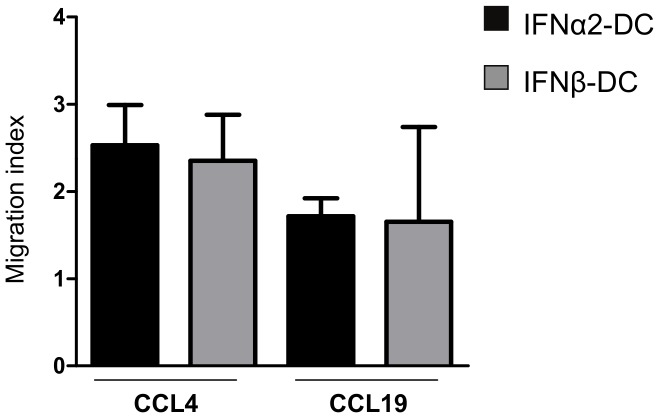
IFNα2-DC and IFNβ-DC migrate comparably in response to CCL4 and CCL19. Chemotaxis assay was performed on IFN-DC in presence of 500 ng/ml CCL4 or 100 ng/ml CCL19. After 2 hours, cells that have migrated through the membrane were counted under the microscope. Error bars represent SEM. Data are from three independent blood donors. The migration index was calculated as the number of cells that migrated toward the chemokine gradient divided by the number of cells that migrated toward the medium alone.

The migration ability of DC is an important characteristic of DC that shapes their functional activities. IFN-DC have been described to exhibit a strong migratory response to specific chemokines such as CCL4 and CCL19, which bind to CCR5 and CCR7 respectively [23], [24]. Thus, we compared the response of IFNα2-DC and IFNβ-DC to these chemokines. Fig. 6 shows that the migration response of both IFNα2 and IFNβ-DC towards CCL4 and CCL19 is similar.

**Figure 7:**
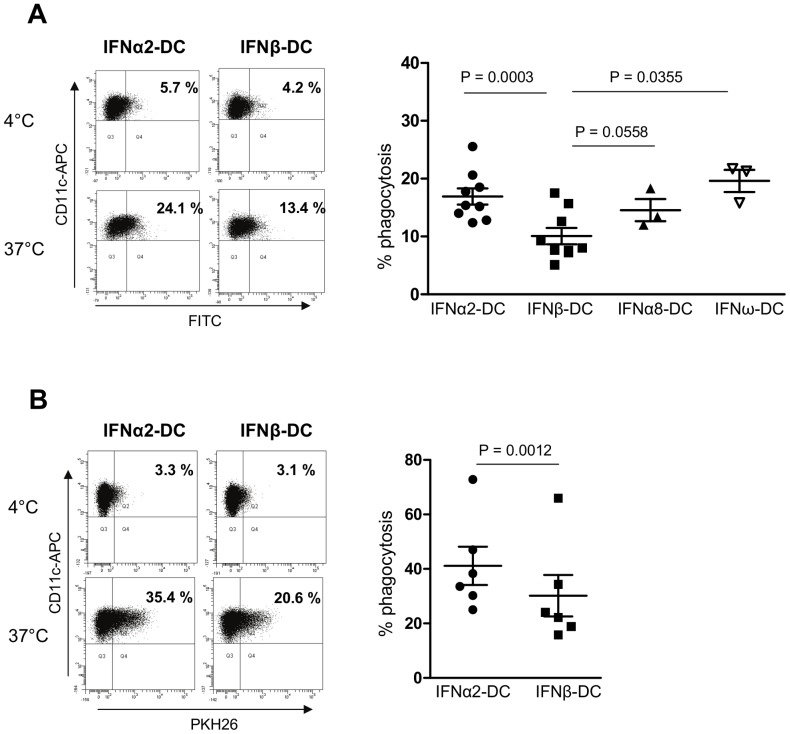
IFNα2-DC are more potent than IFNβ-DC in phagocytosis of apoptotic and necrotic cells. **A.B**. Dendritic cells were cocultured with apoptotic CellTracker®Green-labeled (**A**) or necrotic PKH26-labeled (**B**) LY28 cells for 4 h and then analyzed by FACS. The percentage of phagocytosis was calculated as described in Materials and Methods. In left panels, one representative experiment is shown. Results in right panel A, are from nine experiments carried out on samples from seven independent blood donors. Results in right panel B are from six experiments carried out on five independent blood donors. Error bars indicate SEM. p value was calculated using Student's t-test.

**Figure 8:**
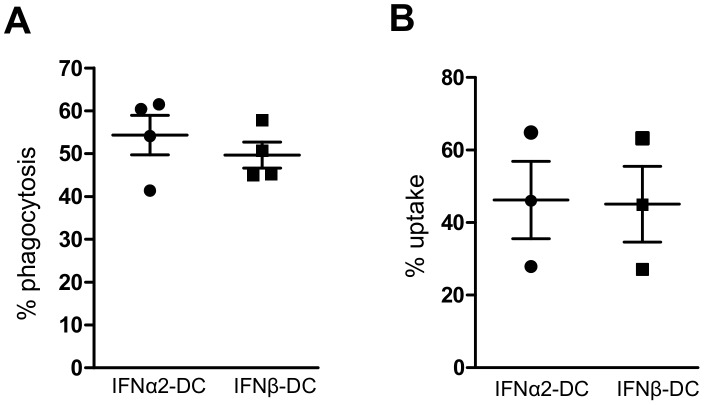
IFNα2-DC and IFNβ-DC are equipotent for the uptake of latex bead or dextran-FITC. **A.B**. IFN-DC were cultured with yellow-green fluorescent latex bead for 4 h (**A**) or with dextran-FITC for 1 h (**B**) before being analyzed by FACS. The percentage of internalization was defined as the percentage of green-labeled-DC at 37°C minus the percentage of green-labeled-DC at 4°C. Experiments were performed on three (B) or four (A) independent donor samples. Error bars indicate SEM.

Figure 7 shows that IFNα2-DC are slightly more potent than IFNβ-DC for the phagocytosis of both apoptotic (16.92%±1.41 and 10.07%±1.41 respectively) and necrotic (41.15%±7.03 and 30.18%±7.59 respectively) cells (Fig. 7A and B). Similarly, IFNα8-DC (14.57±1.91) and IFNω-DC (19.60±1.90) appeared to be more potent than IFNβ-DC in phagocytosis of apoptotic cells, confirming the fact that IFNα-DC and IFNω-DC are closely related and all together distinct from IFNβ-DC. The engulfment of dextran-FITC, which occurs through macropinocytosis, a non specific mechanism, was similar between the two types of IFN-DC (46.23±10.65 and 45.08±10.44 for IFNα2-DC and IFNβ-DC respectively; Fig. 8B), indicating that the difference observed for apoptotic and necrotic cells is specific for phagocytosis. Likewise, the phagocytic uptake of inert latex beads was identical for both IFN-DC samples (54.35±4.61 and 49.68±3.01 for IFNα2-DC and IFNβ-DC respectively; Fig. 8A). These data suggest that the difference observed in the phagocytosis capacity of IFNα2-DC and IFNβ-DC is not due to difference in phagosome membrane synthesis but, at least in part, to the expression of cell surface receptors which are involved in the interaction between apoptotic/necrotic cells and phagocytes and in the recognition of soluble factors secreted by the apoptotic/necrotic cells [25].

## Discussion

The therapeutic antiviral activity of type I IFN is linked to the induction/activation of numerous intracellular signalling pathways that lead to the inhibition of virus replication and also to the stimulation of cells of the immune system, notably T cells, B cells and DC. In particular, type I IFNs are critical for the generation of an antiviral CD8^+^ T cell response, the effectiveness of which depends on the timing and magnitude of type I IFN production [26]. Type I IFN promotes priming of CD8^+^ T cells, notably by enhancing the capacity of DC to process and present viral antigens [27], [28]. Further evidence emphasizes the importance of DC in the antiviral and antitumor effect of IFN. Indeed, Le Bon et al. [29] showed that, *in vivo*, the enhancement of the antibody response against a soluble protein and the increasing production of all subclasses of IgG induced by type I IFN occurs through stimulation of DC. Furthermore, mice lacking IFNAR1 in DC are unable to reject highly immunogenic tumor cells [3].

Type I IFN markedly promotes the differentiation of human blood monocytes into dendritic cells that exhibit strong *in vitro* and *in vivo* capacity to induce a cytotoxic response and cross-priming of CD8^+^ T cells against exogenous viral or tumor associated antigens [15], [30], [31]. Of interest, populations of DC resembling IFN-DC have been observed in patients suffering of autoimmune or infectious diseases, indicating that DC generated *ex-vivo* with type I IFN may indeed resemble naturally occurring DC [32], [33].

In the human system IFNβ can be distinguished from the α and ω subtypes for its higher potency in biological activities that need long term (days) stimulation in the presence of these cytokines, such as inhibition of cell proliferation and osteoclastic differentiation [5], [6]. The differential aspect of these IFN activities is largely independent of early signalling events and early transcriptional response initiated by the formation of the IFN-receptor complex and rather relies, at least in part, on the affinity of IFNβ for its receptor and its ability to escape the negative feedback loop which specifically dampens IFNα activities [11].

In this study, we wished to establish whether type I IFN subtypes exert differential activities on the differentiation of monocytes into DC. We chose to study IFNα2 and β because these two subtypes are routinely used in clinic for pathologies as diverse as chronic viral infection, cancer or multiple sclerosis [1]. In addition, IFNα1, α8, α21 and ω were included in the study. In the human population, IFNα1 and α8 are encoded by two genes subject to strong purifying selection, while the genes encoding the IFNα21, α2, β and ω are more variable [12]. The potencies of these five IFN subtypes towards activation of the Jak/Stat signalling pathway and the ensuing activation of the first wave of ISG transcription varies by a factor 42. Therefore, the concentration of the individual IFN used to differentiate monocytes into DC were standardized relative to 1 nM IFNα2 so that each IFN induced the same initial signal strength and the phenotypes of the IFN-DC generated reflected actual IFN differential activity exerted during the differentiation process.

The transcriptome analysis presented in this study clearly indicates that IFNβ drives a DC differentiation program different from that induced by IFNα1, 2, 8, 21 and ω (Fig. 1 and 2). These two differentiation states were then investigated by assessing expression of several chemokines and cell surface markers important for T cells stimulation. Indeed, IFNβ derived-DC exhibit higher expression of CD80 and CD86, known to be crucial for interaction and activation of T cells, and CD83 (Fig. 3 and Table 2), a marker of activated DC which has been described to inhibit DC-mediated T cell proliferation [34]. Furthermore, IFNβ-DC produce higher amounts of CXCL11 and CXCL10, two chemokines implicated in the attraction of CD8 T cells [35].

**Table 2:** RT-qPCR primers sequence.

Gene	Primer sequence
CCL19	F: 5′-ATGCTGAAGACTGCTGCCTG-3′
	R: 5′-GTCTCTGGATGATGCGTTCT-3′
Clec5A	F: 5′-TACCATCGTGAAGAGAAAAG-3′
	R: 5′-TCATTTGGCATTCTTCTCAC-3′
CXCL5	F: 5′-CGCTGGTCCTGCCGCTGCTG-3′
	R: 5′-ATTTCCTTCCCGTTCTTCA-3′
CXCL11	F: 5′-CGATGCCTAAATCCC-3
	R: 5′-CACAAAACCATAGAAAAGTC-3′
DEFB1	F: 5′-CCTGCCAGTCGCCATGAGAA-3′
	R: 5′-CATTGCCCTCCACTGCTGAC-3′
FOXP3	F: 5′-CCAGGACAGGCCACATTTCA-3′
	R: 5′-CACTGGGATTTGGGAAGGTG-3′
GAPDH	F: 5′-ACAGTCCATGCCATCACTGCC-3′
	R: 5′-GCCTGCTTCACCACCTTCTTG-3′
GATA3	F: 5′-TCAGACCACCACAACCACAC-3′
	R: 5′-CACTTTTTGGATTTGCTAGA-3′
IDO1	F: 5′-AAATGCAAGAACGGGACACT-3′
	R: 5′-TTGCCTTTCCAGCCAGAC-3′
MARCO	F: 5′-CCAGGGAAGCAAGGAGCCAC-3′
	R: 5′-TTCATGCCCCTGTCCCCTTT-3′
RNase 1	F: 5′-GCCTTCCATCTCTCTCAG-3′
	R: 5′-TGCTGGGGGAACTGTCTG-3′
RORγt	F: 5′-CAAAGCATCCTGGCAAAGC-3′
	R: 5′-CCCCACAGGTGACTCGGTTT-3′
T-bet	F: 5′-CCAGTTCATTGCCGTGAC-3′
	R: 5′-AGGATACTGGTTGGGTAGGA-3′
VSIG4	F: 5′-ATGGATGGCTACCTTGGAGA-3′
	R: 5′-ATGCTCTTGTTGGGATGTCT-3′

The above observations suggest that IFNα2-DC and IFNβ-DC could exhibit different functions.

However, it appears that these differential effects do not translate into a differential potency of IFNα2 and IFNβ-differentiated DC to stimulate the proliferation and the polarization of T cells. Indeed, both DCs are equally efficient in stimulating the proliferation of autologous antigen-specific CD4^+^ and CD8^+^ T cells (Fig. 5) and to polarize allogenic naïve CD4^+^ T cells in a Th1 phenotype (Fig. 4). Moreover, both IFNα2 and IFNβ-DC migrate towards chemokines that determine lymph node homing with the same efficiency (Fig. 6). A recent work by Santini et al. [36] reported a Th17 polarization of naïve CD4^+^ T cells stimulated with anti-CD3-coated beads in the presence of autologous IFNα2-DC in the culture. We did not detect any Th17 polarization by either IFNα2 or IFNβ-DC in our experimental settings (Fig. 4), indicating that, at least *ex vivo*, in addition to the factors synthesized by the DC, the strength and duration of TCR stimulation is decisive to determine the nature of naïve T cells polarization.

The only and rather modest difference in the functionality of IFNα/ω-DC and IFNβ-DC is in their efficacy to take up dead cells (Fig. 7). Indeed, IFNα2-, α8- and ω-differentiated DC were slightly more potent than IFNβ-DC in phagocytosis of apoptotic and necrotic cells. This difference was obtained specifically for the phagocytosis since the two DC were equally efficient in macropinocytosis, measured by uptake of dextran-FITC. The difference in phagocytosis is likely consequent to the expression of cell surface receptors necessary for interaction/communication with apoptotic/necrotic cells. Indeed the engulfment of inert latex beads was similar in IFNα2 and IFNβ-DC. At least 5 proteins, identified as being more expressed in IFNα2-DC compared to IFNβ-DC, are known to be implicated in phagocytosis mechanisms and thus may account for the observed difference. CLEC5A is a C-type lectin receptor and several members of this family have been described to have phagocytic activity [37]; MARCO, a class A scavenger receptor, has been shown to act as phagocytic receptor for pathogenic bacteria and apoptotic cells [38], [39]; VSIG4 (CRIg or Z39Ig), a B7 related protein expressed by macrophages and DC has been recognized as a new complement receptor of the immunoglobulin superfamily, required for phagocytosis of circulating pathogens and C3-opsonized particles [40], [41]. Furthermore, the glycosylphosphatidylinositol-linked plasma membrane glycoprotein CD14 has been previously implicated in the phagocytosis of apoptotic cells by macrophages [42].

In conclusion, this study reinforces the notion that IFNβ is unique within the type I IFN family. However, the differences in functionality of IFNα or IFNω-DC vs. IFNβ-DC are rather modest, suggesting that the differential action of the type I IFN subtypes on DC differentiation is not a crucial criterium to take into account in the choice of the IFN subtype to be used in the clinic for a given pathology.

## Materials and Methods

### Interferons

IFNα1 and IFNα21 were produced in bacteria and purified to homogeneity by reverse phase chromatography as previously described [43]. IFNα2 and IFNω were from Dr. G. Adolf (Ernst Boehringer Institute, Vienna, Austria). IFNα8 was from Ciba Geigy Inc. (Basel, Switzerland). IFNβ was from Biogen Idec Inc. (Cambridge, MA, USA). All the IFN used for the generation of DC were controlled to be endotoxin-free. IFNs were assayed for induction of the ISGF3-driven 6–16 promoter in HL116 cell line [44]. Their potencies, relative to IFNα2, for this activity were 21 (α1), 2.9 (α8), 2.3 (α21), 0.5 (β) and 0.6 (ω). The 95% confidence limits of all assays were inferior to 0.1 log.

### Ethics statement

Blood samples from healthy donors were from the Etablissement Français du Sang (EFS, Montpellier) in accordance with a convention between the CNRS and the EFS (21/PVNT/MTP/CNR13/2010-0030). Cord blood samples were from the Centre des Collections Biologiques Hospitalière of Montpellier (CCBH-M) according a material transfer agreement signed by the CNRS and the CHU of Montpellier. This study was approved by the Comité de Protection des Personnes (CPP, Sud Méditerranée IV). All donors provided written informed consent for the collection of samples and subsequent analysis.

### Preparation of interferon-derived dendritic cells (IFN-DC) and T cells

Peripheral blood mononuclear cells (PBMC) were isolated over ficoll gradient (histopaque-1077, Sigma-Aldrich). Monocytes were purified from PBMC by positive or negative selection using respectively either CD14 MicroBeads human (Miltenyi Biotec) or EasySep human monocyte enrichment kit (StemCell Technologies) according to manufacturer's instructions. Monocytes were then plated at the density of 1.6×10^6^ cells/ml/4 cm^2^ in AIM-V medium (GIBCO) and cultured during 3 days with 50 ng/ml GM-CSF (Peprotech), 2% autologous plasma and one of the six following IFN: IFNα1 (21 nM), IFNα2 (1 nM), IFNα8 (2.9 nM), IFNα21 (2.3 nM), IFNβ (500 pM), IFNω (600 pM), according to their specific activity in inducing the early Jak/Stat signaling pathway and ISGF3 transcription factor assembling.

### Measurement of cell surface markers characteristic of DC differentiation state

Expression of cell surface markers characteristic of DC differentiation state was measured by flow cytometry on a FACS CANTO instrument using the following antibodies: CD14-FITC, CD83-FITC, CD80-PE, CD86-APC, HLA-DR-FITC, HLA-ABC-PE, CD40-APC (BD Biosciences). Results were analyzed using BD FACSDiva software.

### RNA isolation and Affymetrix GeneChip processing

Total RNA was isolated from IFN-DC using the High pure RNA isolation kit according to manufacturer's instructions (Roche Diagnostics, France). RNA quality was assessed on an Agilent 2100 bioanalyzer. 200 ng of total RNA from each sample was prepared for hybridization with Affymetrix HG-U133 Plus 2.0 GeneChip (Santa Clara, CA) according to manufacturer's instructions. The hybridized probes were scanned at a resolution of 3 µm in an Affymetrix Genechip scanner 3000 7G. Affymetrix microarrays were processed in the Microarray Core Facility located in the Institute for Research in Biotherapy (Montpellier, France). Data have been deposited on GEO site and are accessible with accession number GSE40268.

Hierarchical clustering of genes/samples was performed using three metrics (Pearson correlation centered, uncentered or Euclidian). Hierarchical clustering was applied to one axis (array) using the weighted pair-group method with centroid average as implemented in the program Cluster [45]. The results were analysed with Tree View [45]. Microarray data were analyzed according to a previously described procedure [20], which allows on the one hand to normalize the signal variance and on the other hand to calculate the expression ratio between two states. The selection criteria used to determine the genes common to all the IFNα-DC were EV≤3.5 and occurrence = 3. Then, the selection criteria of relevant differentially expressed genes between IFNα-DC and IFNβ-DC were such that the selected genes had to meet the two criteria: EV≥3.5 and occurrence = 2.

### RT-qPCR

Total RNA was extracted as described above. 500 ng to 1 µg of total RNA was reversed transcribed using random primers and the SuperScript II Reverse Transcriptase (Invitrogen). RT products were purified using the QIAquick PCR Purification kit (Qiagen) and analyzed on a LightCycler (Roche). Primer sequences are listed in Table 2.

### Multiplex analysis of chemokines

Chemokines in the cell culture supernatant were simultaneously measured using Luminex xMAP technology (Austin, TX, USA) and commercially available antibody beads, according to manufacturer's recommendations (Human Chemokine 10-Plex panel+IL8 from Invitrogen; Milliplex MAP kit Human I-TAC from Millipore). Briefly, antibody-conjugated beads were incubated with standard solution or diluted samples. Subsequent steps involved washes interspersed by the addition of biotinylated detector antibody and Streptavidin-RPE solution. The fluorescence of the beads was read by the Luminex™ 100 system (Austin, TX, USA). The STarStation software (Applied Cytometry, Sheffield, UK) was used for data acquisition and analysis. The MFI value of the analyte in each well was converted into concentration using the standard curve best fitted by 5 parameter logistics.

### Mixed Lymphocyte Reaction (MLR) experiments

CD4^+^ T and CD8^+^ T cells were purified from PBMC using negative selection kits from StemCell technologies and according to manufacturer's protocol. Purity, assessed by flow cytometry, was at least 95%. Naïve CD4^+^ T cells were purified from cord blood units as described above for CD4^+^ T cells.

IFN-DC were cultured with 1.10^5^ allogenic CD4^+^ T cells in conical 96-well plates at different DC∶T cell ratios (1∶10; 1∶40; 1∶150) in a total volume of 150 µl/well of AIM-V medium containing 10% FCS. Five days later, 1 µCi of [methyl-^3^H]-Thymidine (GE Healthcare) was added in each well for a further 18 h incubation time. Radioactivity was counted on a β-counter (TopCount NXT, Packard).

Polarization of naïve allogenic CD4^+^ T cells was analyzed by FACS for marker CD4, CCR4, CCR6 and CXCR3 (BD biosciences) after co-culture of 5.10^5^ IFN-DC with 2.10^6^ naïve CD4^+^ T cells (ratio of 1∶4) in 2 ml AIM-V medium containing 1% TGFβ free FCS (a generous gift from Maria Ferrantini, ISS Rome Italy) in 24 well-plates for 6 days. Alternatively, differentiated CD4^+^ T cells obtained after co-culture of naïve CD4^+^ T cells with IFN-DC were sorted by FACS, mRNA extracted and *Tbet, GATA3, RORγt* and *FoxP3* gene expression levels were measured by RT-qPCR.

### Autologous T cell proliferation assays

IFN-DC were pulsed with recombinant Influenza A virus H1N1 hemagglutinin (A/New Caledonia/20/1999; Sino Biological Inc. China) at 10 µg/ml for 24 h. PBMC-derived CD4^+^ or CD8^+^ T cells were labelled with 1 µM CFSE (Invitrogen) as described [46] and 3.10^5^ T cells were cultured with 6.10^4^ IFN-DC (ratio of 1∶5) in 96-well plates in a final volume of 150 µl in AIM-V medium containing 5% human AB serum. Five days later, T cells were labeled with either anti-CD4-APC-Cy^7^or anti-CD8-APC-Cy^7^ (BD Biosciences) and T cell expansion was evaluated as CFSE dilution by FACS.

### Chemotaxis assays

Polycarbonate transwell inserts for 24 well-plates and with 0.8 µm pore size were used (Corning). 5.10^5^ IFN-DC were placed in the upper compartment in a final volume of 100 µl AIM-V medium. 500 µl AIM-V medium containing or not (control) the chemokine CCL4 (500 ng/ml) or CCL19 (100 ng/ml) were placed in the lower chamber. After 2 hours cells that migrated through the filter were counted under the microscope. Migration index was calculated as the number of cells that migrated toward the chemokine gradient divided by the number of cells that migrated toward the medium alone.

### Phagocytosis and dextran uptake assays

LY28 lymphoblastoïd B cells were labeled with 0.1 µM of cell tracker green CMFDA (Molecular probes) according to manufacturer's instructions and irradiated at a dose of 0.005 J/cm^2^ UVC (254 nm) in a BIO-LINK® BLX (Fisher Scientific) in order to induce apoptosis. Apoptosis was controlled by annexinV-APC/propidium iodide double labeling. FACS analysis indicate that 40–50% of cells were apoptotic. Alternatively, LY28 were labeled with 10 µM PKH26 (Sigma-aldrich) according to manufacturer recommendations and then submitted to three freeze/thaw cycles to produce necrotic cells. IFN-DC were cultured with either apoptotic or necrotic LY28 cells at a ratio of 1∶2 for 4 h at 4°C (control) or 37°C. DC were then labeled with anti-CD11c-APC (BD Biosciences) and analyzed by FACS. Percentage of phagocytosis is defined as the percentage of double labeled DC (either CD11c-APC^+^/cell tracker green^+^ or CD11c-APC^+^/PKH26^+^) at 37°C minus the percentage of double labeled DC at 4°C.

To measure the uptake of latex beads and dextran, IFN-DC were cultured with either latex beads (FluoSpheres carboxylate-modified microspheres, 1 µm, yellow-green fluorescent; Molecular probes) at a ratio of 50 beads for 1 DC for 4 h or 1 mg/ml dextran-FITC (MW 70000, anionic, Molecular probes) for 1 h. Experiments were performed at 4 and 37°C. Percentage of internalization was defined as the percentage of green-labeled-DC. Dead cells were excluded by using propidium iodide.
